# Transcriptomic Profiling Reveals Lysine-Mediated Proliferative Mechanisms in Mongolian Horse Myogenic Satellite Cells

**DOI:** 10.3390/ani15121711

**Published:** 2025-06-09

**Authors:** Yumeng Liu, Yuanyi Liu, Dongyi Bai, Manglai Dugarjaviin, Xinzhuang Zhang

**Affiliations:** 1 Key Laboratory of Equus Germplasm Innovation, Ministry of Agriculture and Rural Affairs, Hohhot 010018, China; liuyumeng@emails.imau.edu.cn (Y.L.); 13470105913@163.com (Y.L.); baidongyi1983@163.com (D.B.); dmanglai@163.com (M.D.); 2 Inner Mongolia Key Laboratory of Equine Science Research and Technology Innovation, Inner Mongolia Agricultural University, Hohhot 010018, China; 3 College of Animal Science, Inner Mongolia Agricultural University, Hohhot 010018, China

**Keywords:** Mongolian horse, lysine, transcriptomic technology, myogenic satellite cells, PPAR signaling pathway, cell proliferation

## Abstract

Skeletal muscle satellite cells are muscle stem cells that play an important role in the growth, development, and repair of skeletal muscle as well as in the locomotor performance of the animal body. Lysine is the first limiting amino acid and is involved in multiple metabolic pathways in the organism to maintain overall physiological requirements. In this study, Mongolian horse satellite cells were cultured using lysine culture solution at different concentrations, and the proliferative capacity of satellite cells was detected by the cck-8 assay, and the optimal culture concentration was selected. Then, whole transcriptome sequencing technology was used to determine the differential gene expression and regulatory pathways during the proliferation of lysine-cultured satellite cells after 48 h of culture. Our findings revealed that 0.5 mmol/L lysine is the optimal concentration to increase satellite cell activity in equine muscle. The differential genes involved in satellite cell proliferation were mainly enriched in the cAMPsignaling pathway, calcium signaling pathway, and PPAR signaling pathway. Furthermore, upregulation of *PLIN5*, *ACADL*, and *FADS2* and downregulation of *LOC100052888* regulated the expression of the PPAR signaling pathway. 0.5 mmol/L lysine was the optimal concentration to increase satellite cell activity. Lysine can regulate mitochondrial function and lipid metabolism through the PPAR signaling pathway, and promote the proliferation of equine myosatellite cells.

## Simple Summary

This study utilized comprehensive transcriptomic technology to perform an in-depth analysis of the biological functions and regulatory pathways associated with the proliferation of Mongolian horse myogenic satellite cells induced by lysine. The research results demonstrated that the Peroxisome Proliferator-Activated Receptor (PPAR) signaling pathway was significantly enriched during satellite cell proliferation. Additionally, genes related to energy metabolism, as well as mitochondrial fatty acid and cholesterol metabolism, were also found to be significantly enriched. Consequently, it is hypothesized that the proliferation of myogenic satellite cells is regulated by the PPAR signaling pathway. This study provides a crucial theoretical basis for post-injury Mongolian horse rehabilitation and muscle growth and development.

## 1. Introduction

Athletic performance is the most important economic trait of racehorses [1], and the horse’s athletic performance relies mainly on well-developed muscles, with skeletal muscle accounting for 40–55% of the body weight of an adult horse [2]. Skeletal muscle is the protein reservoir of the animal organism; 60% of the body’s protein is stored in skeletal muscle. Amino acids are the basic units that make up proteins, and insufficient intake may lead to a decrease in muscle synthesis [3]. Although, horses and cattle and sheep are herbivores, horses are monogastric animals, which digest and utilize essential amino acids in a manner similar to that of pigs, and unlike ruminants that use rumen microbes to synthesize microbial proteins, essential amino acids must be obtained from the feed in order to maintain the body’s needs. Lysine is the first limiting amino acid in horses, which improves protein absorption in the small intestine, facilitates the transport of intestinal amino acid transporter proteins, improves the rate of protein synthesis [4], and increases the expression of genes related to skeletal muscle development, which in turn promotes skeletal muscle growth.

Factors such as breed of horse, feeding practices, physiological stage, and intensity of exercise affect the metabolism of nutrients such as amino acids [5,6]. Previous studies have shown that the level of lysine in the diet affects protein turnover. Currently, lysine deficiency in diets has been found to reduce body size and body weight in yearling horses in animal studies in horses [7]. However, in studies on Thoroughbreds and Quarter Horses, it was found that body requirements could be maintained by feeding normal levels of lysine in the diet (NRC recommendations) [7], and in another study, it was shown that geldings were satisfied by adding twice the NRC-recommended amount of lysine to the diet [8]. Previous studies have shown unequivocally that lysine promotes satellite cell proliferation through the JAK2-STAT3 pathway, regulates myotube differentiation through the Wnt/Ca^2+^ pathway, and reduces cell death through inhibition of apoptosis, which promotes skeletal muscle growth and development [9,10,11]. Lysine deficiency promotes muscle protein degradation through the upregulation of ubiquitin-related genes, and also leads to an increase in intramuscular fat, which leads to autophagy and susceptibility to degradation of myofibrillar proteins in susceptible mouse muscles, whereas lysine excess reduces catabolism through inhibition of genes such as *UBE2D3* [12,13,14]. Therefore, the addition of lysine to equine diets and the mechanism of regulating skeletal muscle growth and development have not been studied. Therefore, this experiment took equine skeletal muscle satellite cells as the research object to explore the concentration effect of lysine on the proliferation process of equine skeletal muscle satellite cells, and to elucidate the regulatory mechanism of lysine in regulating the proliferation of equine skeletal muscle satellite cells by using whole-transcriptomics technology, so as to provide a theoretical basis for the promotion of equine skeletal muscle synthesis and the improvement of equine muscle quality.

## 2. Materials and Methods

### 2.1. Sample Source and Preservation

Semitendinosus samples from 6-month-old Mongolian horses were selected in this study. All procedures were in accordance with the regulatory standards of the Experimental Animal Welfare and Ethics Committee of Inner Mongolia Agricultural University (No. NND2023019). A 5 g amount of semitendinosus muscle samples was stored in cold saline containing 2% antibiotics for the extraction of the horse muscle satellite cells.

### 2.2. Primary Culture and Preservation of Equine Muscle Satellite Cells

The semitendinosus muscle tissue samples were rinsed with DPBS (Gibco, San Francisco, CA, USA), the connective tissue was removed, and the samples were rinsed again. The tissues were cut into 1 cm^3^ blocks and placed in 1.5 mL centrifuge tubes. They were quickly minced with scissors, and 0.1% type IV collagenase was added. The samples were digested at 37 °C for 40 min, and the digestion was terminated by adding an equal volume of complete culture medium. The samples were then homogenized and passed through 100 μm and 40 μm cell sieves, respectively, and transferred to 15 mL centrifuge tubes for centrifugation. The supernatant was removed, and the cells were resuspended in complete culture medium and seeded in culture dishes containing complete culture medium. They were cultured in a 37 °C, 5% CO_2_ incubator. The purity of the horse muscle satellite cells was purified to over 90% using the differential adhesion method. When the cell confluence reached over 90%, the cells were digested with preheated trypsin for 2–3 min, and the digestion was terminated by adding twice the volume of termination solution. The culture medium and cells were collected in a centrifuge tube and centrifuged at 1500 rpm for 5 min. The supernatant was discarded, and 1.5 mL of cryopreservation solution was added. The suspended cells were resuspended in a sterile, enzyme-free cryotube and placed in a −80 °C freezer for 24 h, then transferred to a liquid nitrogen tank for long-term storage.

### 2.3. Cell Thawing

Cells were taken out of liquid nitrogen and rapidly thawed and frozen in 37 °C warm water. One milliliter of culture medium was added to the cells, gently mixed, and transferred to a centrifuge tube. The mixture was centrifuged at 1500 rpm for 5 min. The supernatant was aspirated off, and the cells were gently resuspended in the culture medium and inoculated into culture dishes containing culture medium. The dishes were then incubated in a 37 °C, 5% CO_2_ incubator.

Then, 2.5 Cell Viability Assay2000 cells were inoculated into 96-well plates for overnight incubation and subsequently replaced with lysine-free culture medium (Thermo Fisher Scientific, New York, NY, USA) and starved for 12 h. The lysine-free culture medium was then replaced with lysine culture medium at different concentrations (0, 0.2, 0.5, 1.0, and 2.0 mmol/L) for 24, 48, and 72 h (culture solution formulations (Table A1)). Three replicates were added to each group. Three replicates of each group were incubated with 10 μL of cck-8 assay (Dalian Bogreen Biotechnology Co., Ltd., Dalian, China) for 3 h. Finally, the absorbance (OD) at 450 nm was measured using an enzyme labeling instrument. GraphPad Prism 9.5 software was used for data visualization and two-way ANOVA. *p* < 0.05 indicates a significant difference; *p* < 0.01 indicates a highly significant difference.

### 2.4. Immunofluorescence

The cells were seeded in a 24-well plate where the crawler was placed, and when the cell fusion rate reached 80~90%, the cells were fixed with 4% paraformaldehyde for 40 min, 4% paraformaldehyde was discarded, and washed with DPBS for 10 min/time and 3 times. Then treat with permeabilization solution for 30 min to increase the permeability of cells, discard the permeabilization solution, wash with DPBS, 10 min/time, and wash 3 times. Then, the blocking solution was blocked at room temperature for 1 h, the blocking solution was discarded, and the cleaning solution was used for 10 min/time, washed 3 times, after adding the primary antibody (PAX7) (Developmental Studies Hybridoma Bank, Iowa City, IA, USA) to 4 °C overnight, the primary antibody was added and incubated overnight at 4 °C, the cleaning solution was used for 10 min/time, and the washing solution was washed 3 times, and the secondary antibody(Invitrogen, Carlsbad, CA, USA) was replaced and incubated at room temperature for 1 h, and the cleaning solution was used for 10 min/time, and the washing was washed 3 times. After staining with DAPI for 15 min, wash with cleaning solution for 10 min/time, wash 3 times, remove the crawl slide and place it on a glass slide, which was then observed and recorded under the BX 53 system microscope.

### 2.5. Experimental Design

Seed the same number (50,000) of passage 3 cells into a 10 cm dish containing complete culture medium, overnight, change to lysine-free and serum-free culture medium, and starve for 12 h. Then, they were randomly divided into control group (CON group) and lysine group (Lys group), and the culture medium containing 0 mmol/L lysine and 0.5 mmol/L lysine (optimal concentration) was changed to 48 h at 37 °C and 5% CO_2_ incubator. At the end of treatment, all cells were collected and stored at −80 °C for subsequent assay analysis. There were 5 biological replicates in each group to increase statistical confidence.

### 2.6. RNA Extraction

Total RNA was extracted using the mirVana™ miRNA ISOlation Kit (Thermo Fisher Scientific, New York, NY, USA). A 600 μL amount of Lysis/Binding Buffer was added to the cell samples and mixed well. Then, 30 μL miRNA Homogenate Additive was added and mixed again. The samples were incubated on ice for 10 min. One-fold volume of chloroform was added, and the samples were centrifuged for 5 min. The supernatant was collected. A 1.25-fold volume of ethanol was added and the samples were centrifuged in the column at room temperature for 30 s. The supernatant was discarded. A 10 μL amount of DNase I and 70 μL Buffer RDD QIAGEN mixture was added to the column membrane at room temperature for 15 min. Then, 350 μL miRNA Wash Solution 1 was added, and the samples were centrifuged at 13,000 rpm for 30 s. The column was collected and washed twice with 500 μL Wash Solution 2/3. The samples were centrifuged at 13,000 rpm for 30 s, and the supernatant was discarded. A 100 μL amount of 95 °C preheated Elution Solution was added, and the samples were placed at room temperature for 2 min. The liquid in the tube was collected after centrifugation for 20 s and stored at −80 °C. The total RNA quantity and integrity of the RNA were evaluated using Nanodrop 2000 (Thermo Fisher Scientific, New York, NY, USA) and Agilent 2100 Bioanalyzer (Agilent Technology, Santa Clara, CA, USA).

### 2.7. Library Construction

A 1 μg amount of total RNA was removed and RNA fragmentation was performed using TruSeq Stranded Total RNA with Ribo-Zero Gold (Illumina, San Diego, CA, USA), TruSeq Stranded Total RNA LT—(with Ribo-Zero Plant) (Illumina, San Diego, CA, USA) to remove rRNA and RNA fragmentation, and using RNA fragmentation as a template, the first cDNA strand was synthesized using SuperScript II Reverse Transcriptase (Invitrogen, Carlsbad, CA, USA) and specific markers were introduced, followed by the addition of 5 μL of End Repair Control and 20 μL Second Strand Marking Master Mix were added to synthesize the second cDNA strand to form a double-stranded cDNA, which was purified by AMPure XP magnetic beads. Then, adenine and junction ligation were added to the 3’ end of the cDNA, and AMPure XP magnetic bead purification was performed. A 5 μL amount of PCR Primer Cocktail and 25 μL of PCR Master Mix were added, PCR amplification was performed to enrich the DNA fragments, and the amplification was purified again with AMPure XP magnetic beads. A 1 μL amount of the sample was taken and tested with an Agilent 2100 Bioanalyzer (Agilent Technologies, Santa Clara, CA, USA), and the length and quality of the library were confirmed based on the results to ensure that the library was suitable for subsequent sequencing experiments.

A 1 μg amount of total RNA was taken to construct a small RNA library using the NEB Next Small RNA Library Prep Set for Illumina kit (NEB, Ipswich, MA, USA).

### 2.8. Real-Time Fluorescence Quantitative PCR

Total RNA was extracted from satellite cells by Trizol method, and the concentration and purity of RNA were detected by microplate reader. cDNA was synthesized by TaKaRa PrimeScript™ RT reagent Kit (TaKaRa Bio Inc., Dalian, China). Quantitative real-time PCR was continued using the TaKaRa fluorescent PCR kit TB Green^®^ Premix Ex Taq™ II (RR820A). Reverse transcription was performed at 37 °C for 15 min (reverse transcription) and 85 °C for 5 sec (the inactivation reaction of the reverse transcriptase), 4 °C. The PCR reaction system was TB Green Premix Ex Taq Ⅱ12.5 μL, upstream and downstream primers (10 μmol/μL) 1.0 μL, DNA template 2.0 μL, and sterilized water 8.5 μL, respectively. The assay was performed on a CFX96 PCR apparatus (BIO-RAD, Hercules, CA, USA). PCR was performed at 95 °C for 30 s. A total of 39 cycles of 95 °C for 5 s followed by 60 °C for 30 s were performed. Using a Primer (5.0) software, according to the NCBI reference sequence design primers (https://www.ncbi.nlm.nih.gov (accessed on 6 November 2024)). *GAPDH* was used as the reference gene, and the expression levels of *CSPG4*, *KIF2C*, *MATK*, *TPX2*, *INPP5D*, *PPID*, *UNC5B*, and *LOC100069322* genes were detected to verify the accuracy of the whole transcriptome. *ACADL*, *PLIN5*, *SCD5*, and *FADS2* were used to verify mitochondrial function. *PAX7*, *KI-67*, and *MYOD* were used to verify cell proliferation, and the relative expression of genes was calculated by 2^−∆∆Ct^ method. The difference in data was analyzed by *t* test. *p* > 0.05 was not significant, and *p* < 0.05 was significant. The initial information is presented in Appendix A, Table A2.

### 2.9. GO and KEGG Enrichment Analysis of Dif-mRNA, Dif-lncRNA, Dif-circRNA, and Dif-miRNAs

Differential transcripts with *p*-value < 0.05 and |log2 (fold change)| ≥ 2 were used to calculate the significance of differential gene enrichment in each Gene Ontology (GO) and Pathway entry by applying the hypergeometric distribution algorithm, and the results were tested for accuracy using the *p*-value by Benjamini and Hochberg’s multiple test.

### 2.10. Competing Endogenous RNA (ceRNA) Network Analysis

Based on the test results, with differential mRNA, lncRNA, circRNA, and miRNA genes as the core, the function-related differential genes were queried based on GO function enrichment analysis and Kyoto Encyclopedia of Genes and Genomes (KEGG) pathway enrichment analysis, and the relationship pairs of all three of lncRNA-miRNA-mRNA, and all three of circRNA-miRNA-mRNA were extracted that were plotted using Cytoscape 3.9.1 [15]. The Pearson correlation coefficients were generated by calculating the expression matrix data of mRNA, lncRNA, and circRNA. The correlation analysis was set with the threshold of the absolute value of the correlation coefficient ≥ 0.90 and *p* < 0.05 to obtain the co-expression relationships between dif-mRNA-dif-lncRNA and dif-mRNA-dif-circRNA.

### 2.11. Mitochondrial Stress Detection

**Figure A1:**
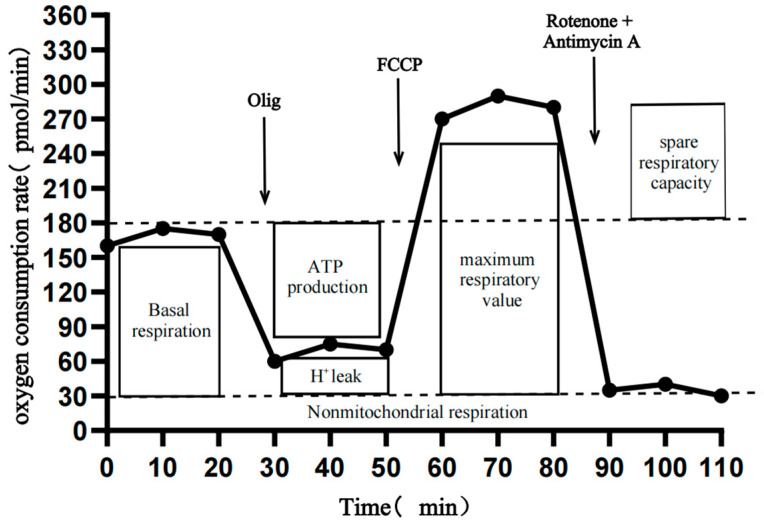
Seahorse XFp assay mitochondrial function standard curve.

After processing, the cells were detected in real time for mitochondrial oxygen consumption rate (OCR) using XFp (Seahorse XFp Analyzer (Agilent Technologies, Santa Clara, CA, USA)). After determining the basal OCR, 2.5 μmol/L oligomycin (Olig, New York, NY, USA), 2.0 μmol/L uncoupling agent of oxidative phosphorylation (carbonyl cyanide-4-(trifluoromethoxy)phenylhydrazone, FCCP), and rotenone, antimycin A mixture were continuously injected. The mitochondrial respiratory values (ATP consumption values), reserve respiratory (the difference between the maximum respiratory value and the basal respiratory value), maximum respiratory, and non-mitochondrial respiratory were obtained, respectively. Proton leakage was the basal respiratory value minus the ATP consumption value (Appendix A, Figure A1). The mitochondrial stress parameters were analyzed using *t*-test. *p* > 0.05 indicated no significant difference; *p* < 0.05 indicated significant difference; *p* < 0.01 indicated extremely significant difference.

## 3. Results

### 3.1. Effect of Lysine on the Viability of Mongolian Horse Myosatellite Cells

**Figure 1:**
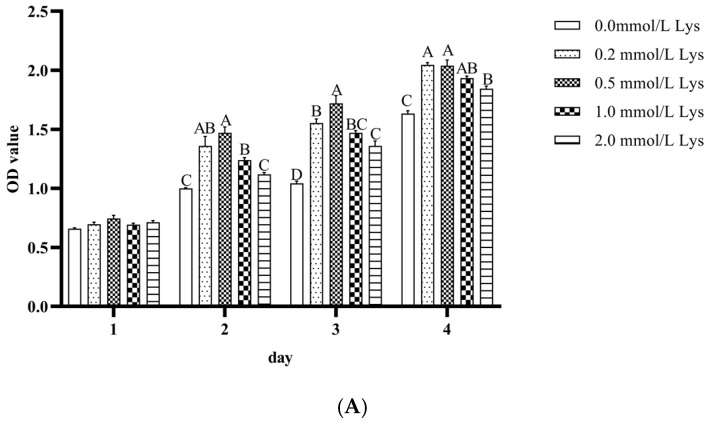

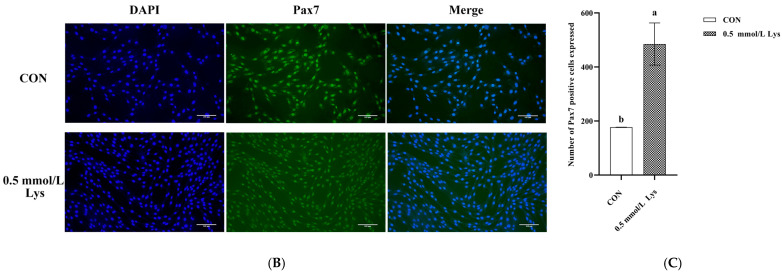
Effects of lysine on satellite cell proliferation. (**A**) cck-8 assay was used to detect the effect of different concentrations of lysine on the viability of muscle satellite cells; (**B**) Immunofluorescence was used to detect cell proliferation; (**C**) Analysis of immunofluorescence results. The immunofluorescence scale was 100 μm. Different lowercase letters indicate significant differences (*p* < 0.05), different uppercase letters indicate highly significant differences (*p* > 0.01), and the same uppercase and lowercase letters indicate non-significant differences (*p* > 0.05).

As can be seen from Figure 1, the effects of different lysine concentrations on Mongolian horse myosatellite cell proliferation. The cck-8 assay showed that the proliferative effect of lysine on Mongolian horse myosatellite cells showed a dose effect, and there was no significant difference between each group after 24 h (1 d) of cell culture (*p* > 0.05). Significant differences were detected in the proliferative effects of Mongolian horse myosatellite cells at 48, 72, and 96 h (*p* < 0.05). At 48 h and 72 h, the proliferative effects of 0.5 mmol/L lysine were not significantly different from the proliferative effect of Mongolian horse myosatellite cells of 0.2 mmol/L and 1.0 mmol/L lysine, respectively (*p* > 0.05), and were significantly different from all other groups (*p* < 0.05). After 96 h, the proliferative effect of 0.5 mmol/L lysine was significantly higher than that of CON and 2.0 mmol/L lysine. The number of PAX7-positive equine myosatellite cells was significantly higher in the 0.5 mmol/L lysine group than in the control group (*p* < 0.05) (Figure 1B,C). Therefore, 0.5 mmol/L lysine had the best effect on the proliferation of Mongolian horse myosatellite cells, which can be used for subsequent investigation of the mechanism of lysine regulation on the proliferation of Mongolian horse myosatellite cells using whole transcriptome technology.

### 3.2. Lysine Mediates Differential Gene Expression in Mongolian Horse Myosatellite Cells

**Figure 2:**
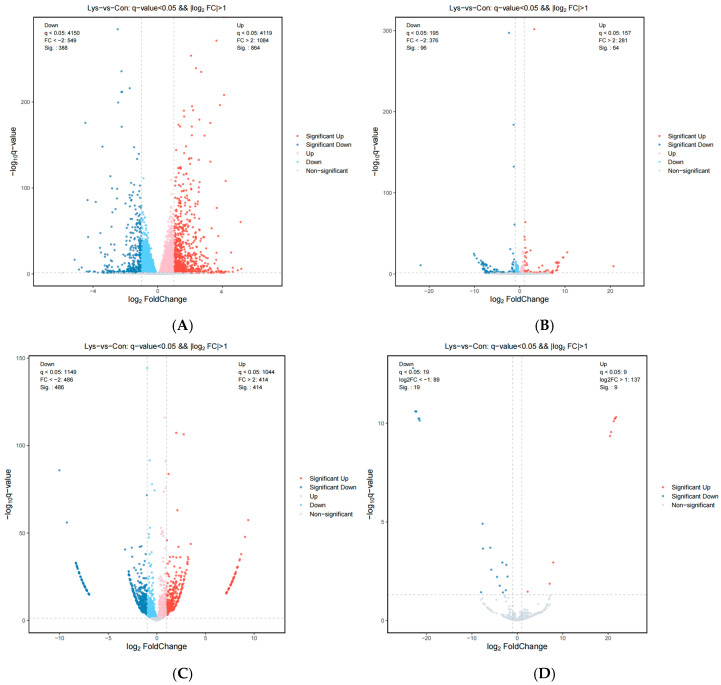
The effect of lysine on the expression of differential genes in Mongolian horse muscle satellite cells. (**A**) mRNAs differential gene volcano plot; (**B**) lnRNAs differential gene volcano plot; (**C**) circRNAs differential gene volcano plot; (**D**) miRNAs differential gene volcano plot.

**Figure 3:**
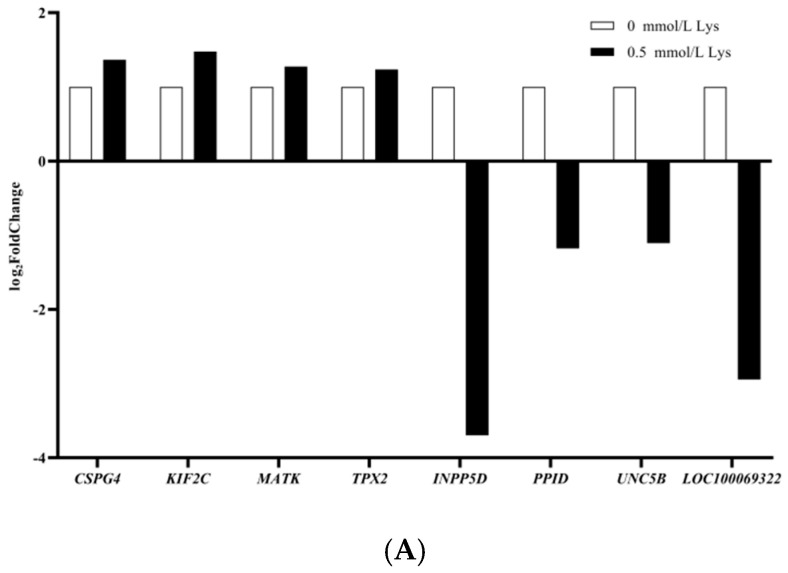

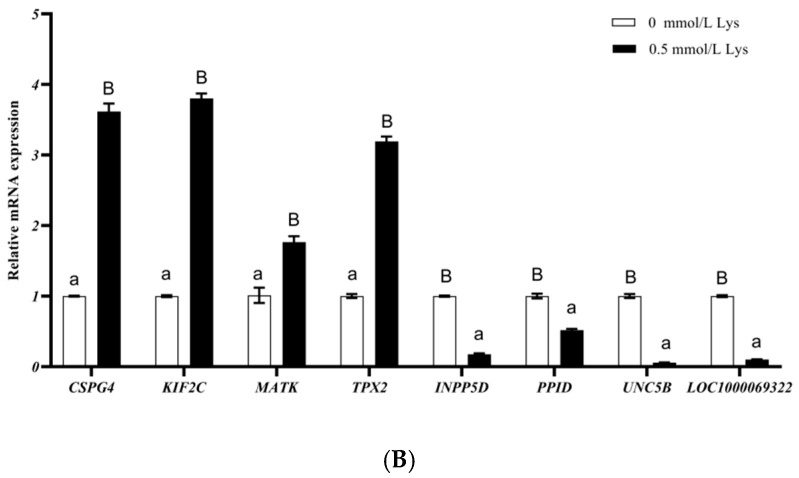
Histograms of RNA−Seq and RT−qPCR. (**A**) RNA-Seq graph; (**B**) RT−qPCR gene validation graph. The same lowercase letter is for a non-significant difference (*p* > 0.05), different lowercase letters are for a significant difference (*p* < 0.05); different forms of letters are for a highly significant difference (*p* < 0.01).

**Figure A2:**
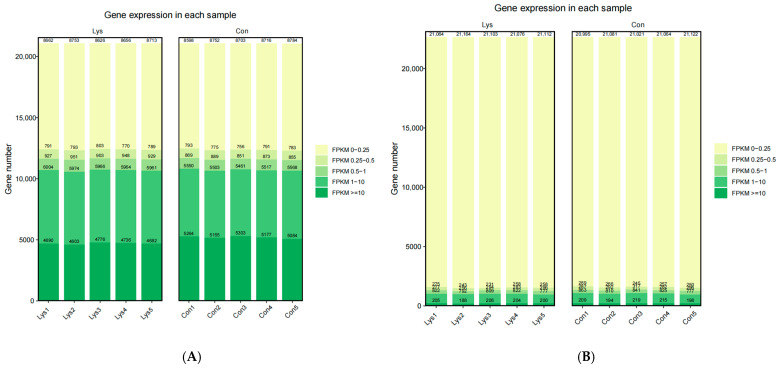

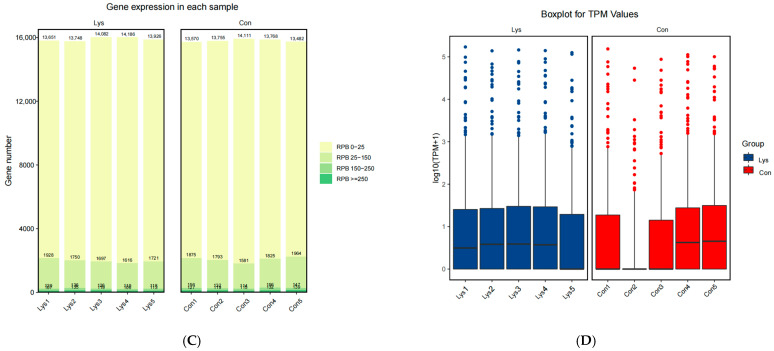
The effect of lysine on the expression of differential genes in Mongolian horse muscle satellite cells. (**A**) Distribution of FPKM expression for mRNAs; (**B**) Distribution of FPKM expression for lnRNAs; (**C**) Distribution of RPB expression of cicrRNAs; (**D**) Line box plot of TPM expression of miRNAs.

After lysine treatment, the experiments used volcano plots to represent the differential expression of mRNAs, lnRNAs, circRNAs, and miRNAs, while FPKM (fragments per kilobase of exon model per million mapped fragments) was used to calculate the FPKM expression distribution of mRNAs, lnRNAs, and RPB (junction reads per billion mapped reads) for quantification of circRNA, miRNA expression was calculated using TPM (TPM). The FPKM distribution of mRNAs, lnRNAs was calculated using FPKM (fragments per kilobase of exon model per million mapped fragments) method, circRNAs were quantified using RPB (junction reads per billion mapped reads), and miRNAs expression was calculated using TPM (transcript per million) (Appendix A, Figure A2A–D), and the *p*-value and multiple changes were listed (Supplementary Table S1). The differential expression conformed to the following rules: *p* < 0.05 and log2 (multiple change) > 2. A total of 1252 differentially expressed mRNAs, 176 differentially expressed lncRNAs, 900 differentially expressed circRNAs, and 28 differentially expressed miRNAs were found in Mongolian horse myosatellite cells in the 0 mmol/L and 0.5 mmol/L lysine groups (Figure 2). Of these, 864 mRNAs were upregulated, 388 mRNAs were downregulated (Figure 2A), 73 lncRNAs were upregulated, 103 lncRNAs were downregulated (Figure 2B), 414 circRNAs were upregulated, 486 circRNAs were downregulated (Figure 2C), 9 miRNAs were upregulated, and 19 miRNAs were downregulated (Figure 2D). The differences between the two groups were significant, indicating high reliability of the results of differential expression. Eight different mRNAs were screened to detect the gene expression levels in the two groups using real-time fluorescence quantitative PCR, and the RNA-Seq and RT-qPCR results for all genes were consistent (Figure 3A,B). Volcano and heat maps can be used to visualize the differential expression of mRNAs and ceRNAs.

### 3.3. GO Functional Analysis and KEGG Pathway Analysis of Lysine-Induced Mongolian Horse Myosatellite Cell Differential mRNAs

**Figure 4:**
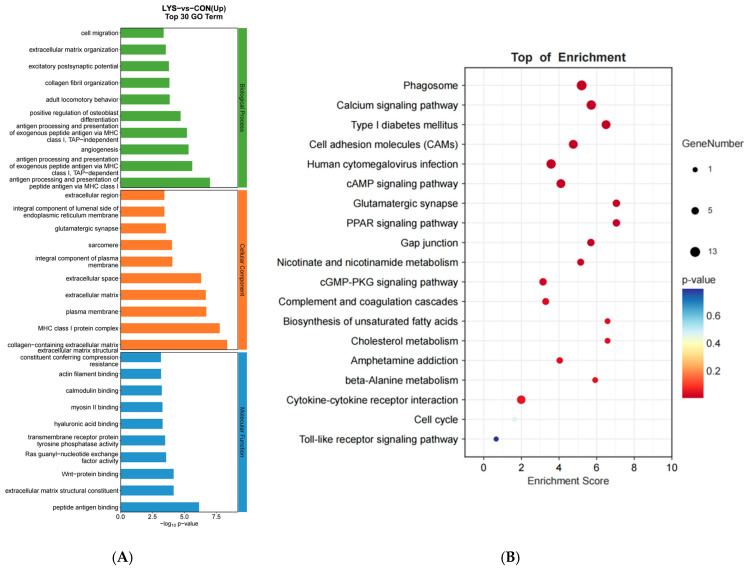
Lysine-induced upregulation of differential mRNAs in Mongolian horse myosatellite cells by GO functional analysis and KEGG pathway analysis. (**A**) GO functional enrichment map of differentially expressed genes in mRNAs; (**B**) KEGG signal pathway enrichment map of mRNAs.

After data analysis, GO functional enrichment analysis of differentially expressed mRNAs showed that, in terms of biological processes, differentially expressed mRNAs were significantly enriched in functions such as cell proliferation, positive regulation of cell migration, plasma membrane repair, regulation of the cell cycle process, mitotic cell cycle, Wnt signaling pathway, cell adhesion, and cell migration. In terms of cellular components, they were mainly enriched in functions such as chromosomes, myofilaments, and condensed chromosome attachment granules. In terms of molecular functions, they were enriched in calcium binding, protein serine/threonine kinase activity, actin binding, and Wnt protein binding (Figure 4A). Significant enrichment analysis of the KEGG pathway showed that differentially expressed mRNAs were significantly enriched in the calcium signaling pathway, cellular adhesion molecules (CAMs), cAMP signaling pathway, the PPAR signaling pathway, biosynthesis of unsaturated fatty acids, cholesterol metabolism, β-alanine metabolism, and cell cycle pathways (Figure 4B).

### 3.4. GO Functional Analysis and KEGG Pathway Analysis of Lysine-Induced Differential lncRNAs and miRNAs in Mongolian Horse Myosatellite Cells

**Figure 5:**
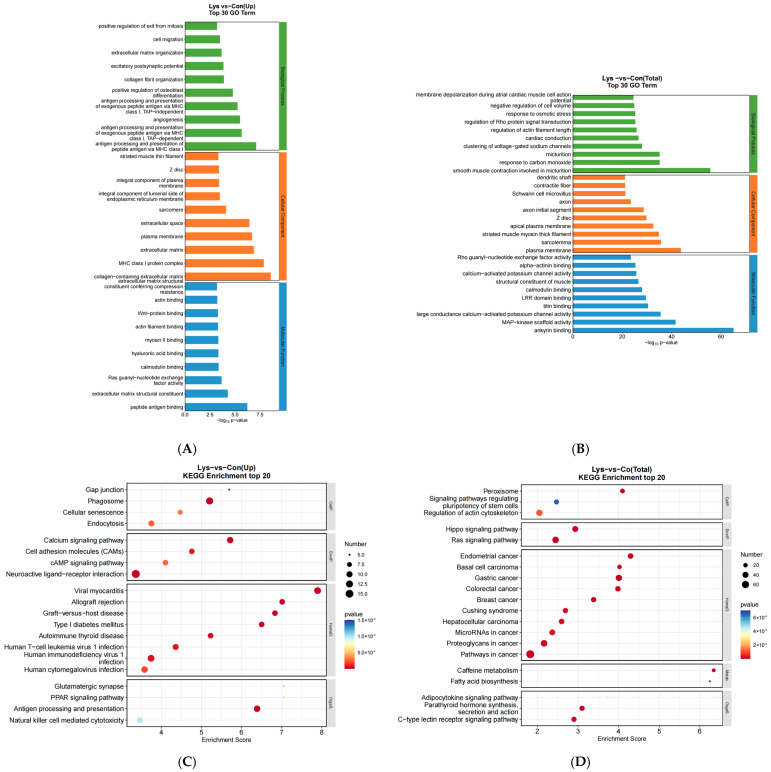
Lysine induces differentially expressed upregulated lncRNAs and miRNAs in Mongolian horse myosatellite cells, GO functional analysis, and KEGG signaling pathway. (**A**) GO functional enrichment map of lncRNAs; (**B**) GO functional enrichment map of miRNAs; (**C**) KEGG signaling pathway enrichment map of lncRNAs; (**D**) KEGG signaling pathway enrichment map of miRNAs.

Co-expression enrichment analysis of differentially expressed mRNAs and lncRNAs was performed using Pearson correlation methods, and GO functional enrichment analysis showed that, in terms of biological processes, co-expression enrichment was analyzed in cell migration, extracellular matrix organization, excitatory postsynaptic function, and antigenic processing and presentation of peptide antigens through the MHCI classes. In terms of cellular components, rhabdomyosin filaments, components of the plasma membrane, myonodules, extracellular space, and MHCI-like protein complexes, and in terms of molecular functions, actin-binding, Wnt-protein-binding, myosin II-binding, and structural components of the extracellular matrix (Figure 5A). Significant enrichment analyses of the KEGG pathway revealed antigen processing and presentation, calcium signaling pathways, graft-versus-host disease, cell adhesion molecules (CAMs), cAMP signaling pathway, the Peroxisome Proliferator-Activated Receptor (PPAR) signaling pathway, gap junctions, and other pathways (Figure 5C).

Differential miRNA target gene GO function and KEGG pathway enrichment analysis, in terms of biological process, the target gene function is mainly enriched in the regulation of actin filament length, aggregation of voltage-gated sodium channels and carbon monoxide response, etc., and in terms of cellular composition, it is enriched in the functions of contractile fibers, Z disc, myosin thick filaments and myofibrillar membranes, etc. (Figure 5B), and in terms of molecular functions KEGG signaling pathway analysis, mainly enriched in peroxisomes, signaling pathways regulating stem cell pluripotency, actin cytoskeleton regulation, Hippo signaling pathway, cancer pathways, fatty acid biosynthesis and dimerization cytokine signaling pathways, and other pathways (Figure 5D).

### 3.5. ceRNA Network Construction

**Figure 6:**
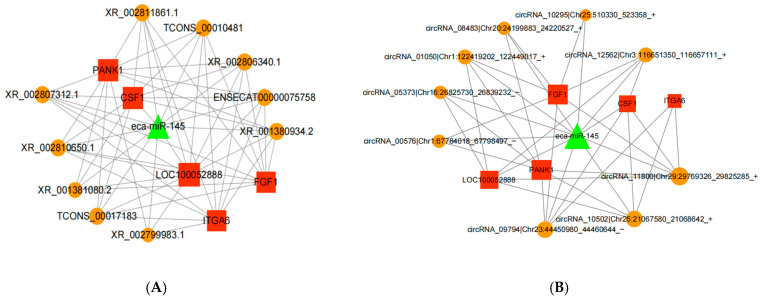
ceRNA network construction. (**A**) miRNA-lncRNA-mRNA network construction; (**B**) miRNA-circRNA-mRNA network construction.

Based on the relationship between gene co-expression and the regulatory relationships of dif-miRNA-dif-mRNA, dif-miRNA-dif-lncRNA, dif-cicrRNA-dif-mRNA, dif-miRNA-dif-cicrRNA, dif-miRNA-dif-cicrRNA, and dif-miRNA-dif-cicrRNA, which were regulated by the same miRNAs, 37 miRNA-circRNA-mRNA interaction pairs were identified, containing five upregulated dif-mRNAs, nine downregulated dif-cicrRNAs, and one up A total of 27 lncRNA-miRNA-mRNA interaction pairs containing five upregulated dif-mRNAs, nine downregulated dif-lncRNAs, and one upregulated miRNA were identified (Figure 6A,B).

**Figure 7:**
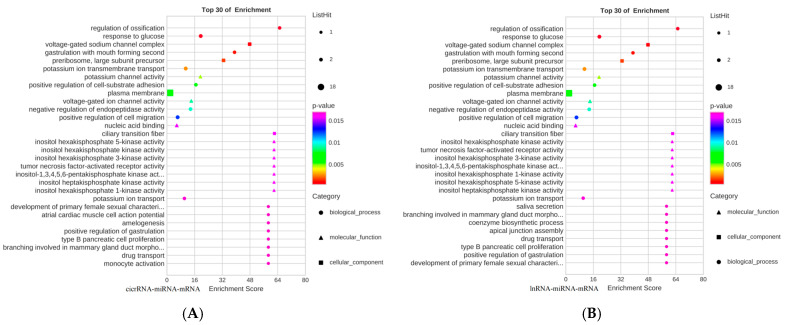

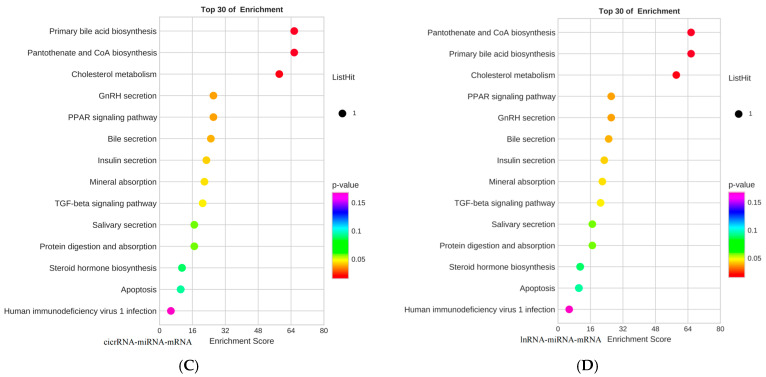
ceRNA regulatory network and GO functional enrichment analysis of miRNA-lncRNA-mRNA and miRNA-circRNA-mRNA and KEGG signaling pathway analysis. (**A**) GO functional enrichment map of miRNA-lncRNA-mRNA; (**B**) GO functional enrichment map of miRNA-circRNA-mRNA; (**C**) KEGG signaling pathway enrichment map of miRNA-lncRNA-mRNA; (**D**) KEGG signaling pathway enrichment map of miRNA-circRNA-mRNA.

The two ceRNA networks were subjected to GO functional enrichment analysis, and their major roles are shown in the analysis plots (Figure 7A,B). And KEGG signaling pathway enrichment analysis was mainly involved in primary bile acid biosynthesis, cholesterol metabolism, PPAR signaling pathway, bile secretion, insulin secretion, TGF-β signaling pathway, and protein digestion (Figure 7C,D).

### 3.6. Lysine Regulates the PPAR Pathway to Provide Energy for the Proliferation of Mongolian Horse Myosatellite Cells

**Figure 8:**
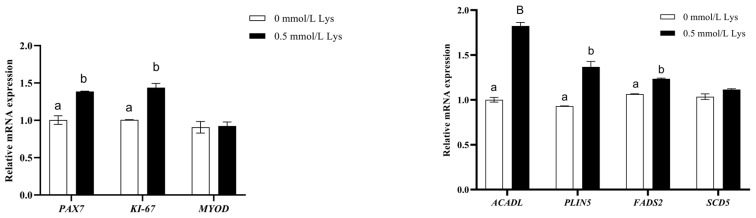

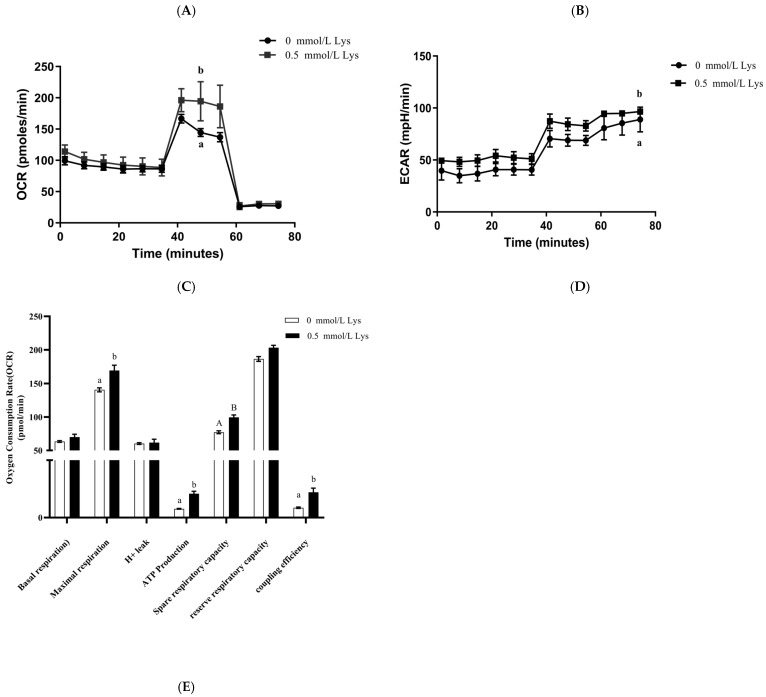
Lysine regulates the PPAR pathway to provide energy for proliferation of Mongolian horse myosatellite cells. (**A**) Expression levels of satellite cell proliferation-related genes mRNA; (**B**) Genes related to the PPAR signaling pathway; (**C**) OCR for mitochondrial stress detection; (**D**) ECAR of satellite cells; (**E**) Mitochondrial stress parameters. Different lowercase letters are significant (*p* < 0.05) and different forms of letters are highly significant (*p* < 0.01).

As shown in Figure 8, the mRNA expression of paired box 7 (PAX7) and antigen identified by monoclonal antibody KI—67 (KI—67) in the 0.5 mmol/L Lys group was significantly higher than that in the 0 mmol/L Lys group (*p* < 0.05) for the proliferation of Mongolian horse muscle satellite cells (Figure 8A). The mRNA expression of long-chain acyl coenzyme A dehydrogenase (ACADL) in the 0.5 mol/L Lys group was significantly higher than that in the 0 mmol/L Lys group (*p* < 0.01), and the mRNA expression of PLIN5 and lipid desaturase 2 (FADS2) in the 0.5 mol/L Lys group was significantly higher than that in the 0 mmol/L Lys group (*p* < 0.01). The Lys group had significantly higher mRNA expression of PLIN5 and FADS2 than the 0 mmol/L Lys group (*p* < 0.05) (Figure 8B), and OCR, ECAR, maximum respiratory value, ATP production, and coupling efficiency were significantly higher in the 0.5 mmol/L Lys group than the 0 mmol/L Lys group (*p* < 0.05) (Figure 8C–E). The spare respiratory capacity of the 0.5 mmol/L Lys group was significantly higher than that of the 0 mmol/L Lys group (*p* < 0.01) (Figure 8E). Therefore, the proliferation of Mongolian horse myosatellite cells may regulate mitochondrial lipid metabolism via the lysine-mediated PPAR signaling pathway.

## 4. Discussion

During the early growth phase of animals, skeletal muscle satellite cells are involved in muscle development with the help of increasing the number of myonuclei and expanding the structural domains of myonuclei, which determines motor performance [16]. External stimuli cause resting satellite cells to enter the cell cycle and promote mitosis, increasing the number of satellite cells. In this study, lysine was found to increase the viability of equine muscle satellite cells, and the best cell viability and proliferation capacity was found at 0.5 mmol/L lysine. This is similar to the results of previous studies, in which the addition of lysine increased satellite cell viability and decreased satellite cell apoptosis, and the lack of lysine inhibited cell proliferation and led to an increase in apoptosis [9,11]. Therefore, 0.5 mmol/L lysine was selected for subsequent experiments to provide a theoretical premise for in-depth investigation of lysine’s effect on the proliferation process of equine muscle satellite cells.

Lysine is the first limiting amino acid, which can promote the proliferation of satellite cells, participate in amino acid transport, and affect protein synthesis in muscle by regulating the expression of myosin, CAT-1 system, or myogenic factors [10,17,18]. Therefore, lysine has an important role in the growth and development of skeletal muscle as well as in athletic performance. In this experiment, we chose the whole transcriptome to analyze the mRNA and ncRNA (miRNA, lncRNA, circRNA) expression profiles, aiming to comprehensively dissect the potential relationship between RNAs and to clarify the mechanism by which lysine regulates the proliferative process of equine myosatellite cells. The analysis revealed that *ABRA*, *FHOD3*, *MYOZ3*, *ACTN3*, and *ACTN1* were upregulated. Actin-binding Rho activating protein (ABRA), also known as myosin-activated Rho signaling protein (STARS), is located in the nucleus or cytoplasm of the cell, and not only has two independent actin-binding domains [19], but also has reciprocal binding sites on its promoter for muscle-specific transcription factors. STARS binds to ABLIM proteins and enhances the serum response factor activity, which regulates the growth process of myocytes [20]. Arai et al. [21] clearly indicated that STARS activates Rho, which in turn promotes actin polymerization. Wallace et al. [22] found that there are two conserved E boxes on the STARS promoter, which act to activate STARS by recruiting MyoD to complete the C2C12 cell differentiation process. In addition to this, previous studies have shown that STARS is also involved in functions such as functional myonodule formation, actin filament binding, and Z disc protein interactions [20,21,22]. This is consistent with the findings of the present study. In this study, we also found that *FHOD3* expression was upregulated by the addition of lysine and was involved in the functions of rhabdomyosin filaments, sarcomeres, actin-binding, and actin-filament binding. Taniguchi et al. [23] showed that FHOD3 is localized to actin filament-forming proteins, which bind directly to the actin filament sub-termini, promote sarcomere formation by activation of serum-responsive factors, and play an important role in regulating the assembly and turnover of actin in sarcomeres. FHOD3 deficiency leads to a reduction in filamentous actin and disruption of myonodule structure. alpha-actinin-1 (ACTN1) is controlled by ATP hydrolysis, ions, and multiple actin-binding proteins to polymerize G-actin to form F-actin. The above results suggest that 0.5 mmol/L lysine can affect actin binding, rhabdomyosin filament, and myonodule function through upregulation of *ABRA*, *FHOD3*, and *ACTN1* genes, thereby promoting myosatellite proliferation, which in turn primes myoblastogenesis and the process of skeletal muscle development. Myosin-binding protein 3 (MYOZ3) is upregulated in the actin-binding and Z disc. MYOZ3 regulates Z disc structure and signal transduction, and interacts with a variety of Z disc proteins to influence the formation and maintenance of Z disc [24], and it is a key gene affecting the type of muscle fiber, mainly expressed in mammalian fast muscle fibers [25], increasing muscle strength, grows muscle size and improves the explosive power of the organism’s movement, among other functions [26]. The horse’s ability to efficiently stimulate Type IIX and IIA/II fibers to contract quickly and adapt to high-intensity training [27]. Also, Alpha-actinin-3 (ACTN3) showed upregulation in the present results. Chan et al. [28] showed that *ACTN3* promotes the differentiation of muscle fibers to fast muscle fibers in mice, and the lack of *ACTN3* leads to a reduction in muscle cross-sectional area, a lower ratio of tonic contraction, and a greater resistance to fatigue. The experimental results showed that 0.5 mmol/L lysine upregulation of *ACTN3* in equine muscle satellite cells may increase the content of fast muscle fibers in the muscle, and *MYOZ3* may increase the rate of muscle contraction, which improves the sprinting performance, explosive power, and short-distance speed racehorse athletic performance [29].

Therefore, lysine regulates actin dynamics, affects cytoskeleton formation, improves myosatellite cell viability, and thus promotes raw muscle fiber development and transformation by upregulating *ABRA*, *FHOD3*, *MYOZ3*, *ACTN1*, and *ACTN*3 genes in the myosatellite cells, acting on the structures of the Z disc, transverse muscle filaments, myofibrils, actin-binding, and myosin-II-binding, and consequently and elevating the horse’s muscle contraction capacity and athletic performance. In KEGG signaling pathway enrichment analysis, the calcium signaling pathway, the cyclic adenosine monophosphate (cAMP) signaling pathway, and the PPAR signaling pathway were annotated after lysine treatment. The calcium signaling pathway regulates the processes of cell proliferation, differentiation, and apoptosis through dynamic changes in Ca^2+^. Changes in intracellular Ca^2+^ can regulate the process of cAMP production and hydrolysis through adenylate cyclase and phosphodiesterase [30].

cAMP acts as an intracellular second messenger and regulates Ca^2+^ dynamics together with its effector proteins, suggesting that lysine can regulate the proliferation of equine myosatellite cells through the interaction of the calcium signaling pathway and the cAMP signaling pathway. KEGG analysis showed that in lysine-treated equine myosatellite cells, the PPAR pathway was significantly activated, and its key genes (*ACADL*, *FADS2*, *PLIN5*) were upregulated, which was directly related to the regulation of fatty acid oxidation and lipid metabolism and became the core of this study. Long-chain acyl coenzyme A dehydrogenase (ACADL) catalyzes the first step of β-oxidation of long-chain fatty acids and is directly regulated by PPARα. Moderate amounts of nitrate increased *ACADL* expression through activation of PPARα, which led to an increase in the rate of oxidative phosphorylation of carnitine palmitate and enhanced mitochondrial respiratory function [31]. Fatty acid desaturase (FADS2) is the first rate-limiting enzyme in the biosynthesis of polyunsaturated fatty acids, mainly acting on α-linolenic acid (n-3) and linoleic acid (n-6), and its expression is regulated by *SREBP1* [32]. Inhibition of *FADS2* downregulated the expression of STAT3 phosphorylated proteins, leading to a decrease in protein content in murine gastrocnemius muscle, an effect that was reversed by n-3 supplementation [33]. Overexpression of *FADS2* increased the concentration of dihomo-γ-linolenic acid and docosahexaenoids in goat mammary epithelial cells [32]. Perilipin 5 (PLIN5) may serve as a metabolic bridge between lipid droplets and mitochondria, accelerating fatty acid transport and preventing oxidative stress in skeletal muscle. Muscle-specific knockdown of *PLIN5* in mice resulted in decreased muscle FGF21 secretion and impeded organismal metabolism [34]. In lipid overload, it leads to incomplete oxidation of mitochondrial FA and produces accumulation of harmful substances, which can regulate lipid metabolism through the PPARα/PGC-1α signaling pathway involved in *ACADL* [35]. *PLIN5* mediates PPARα and PGC1α, increases triglyceride storage, protects mitochondrial function, and reduces lipotoxicity damage [36]. RT-qPCR results showed that lysine treatment significantly upregulated the mRNA expression levels of *ACADL*, *FADS2*, and df in equine myosatellite cells, suggesting that lysine regulates fatty acid oxidation in equine myosatellite cells. The results of this experiment showed that mitochondrial respiratory efficiency was significantly increased in horse myosatellite cells after lysine treatment, which may be related to the fact that *ACADL* enhances palmitic acid oxidation efficiency and supports mitochondrial respiratory chain activity. The increase in ATP production may be mediated by *PLIN5*, mediating lipid–mitochondrial coupling and unsaturated fatty acids by inducing the expression of *ACADL* in the PPARα/PGC-1α signaling pathway, the Improvement of β-oxidation efficiency. Based on the above analysis, lysine may regulate fatty acid oxidation and mitochondrial function through upregulation of *ACADL*, *FADS2*, and *PLIN5* genes in the PPAR signaling pathway, thereby promoting the proliferation of mosaic satellite cells.

In gene regulatory networks, long chain non-coding RNAs (lncRNAs), circular RNAs (circRNAs), and messenger RNAs (mRNAs) can interact with microRNAs (miRNAs), and competing endogenous RNAs (ceRNAs) play key roles in a multitude of biological processes by competing for binding to common miRNAs to construct regulatory relationships. For example, the CTTN-IT1-miR-29a-YAP1 regulatory network promotes proliferation and differentiation of skeletal muscle satellite cells, and the ceRNA network formed by insulin-like growth factor 2 (IGF2BP1) and *miR-145-3p* precisely regulates skeletal muscle satellite cell proliferation [37]. In the present study, we have newly found that after lysine supplementation, the gene *LOC100052888* was involved in the PPAR signaling pathway, but its expression level was downregulated, while *miR-145* expression was significantly upregulated, and the two formed a ceRNA network to jointly regulate cholesterol metabolism. The gene *LOC100052888* encodes cholesterol 7α-hydroxylase (CYP7A1), which is dually regulated by *PPARα* and *PPARγ* in cholesterol metabolism, with *PPARα* inhibiting its expression and *PPARγ* promoting its expression [38,39]. Combined with the discovery of fatty acid oxidation-related genes, it is hypothesized that lysine may mainly supply energy to cells through the lipid metabolism pathway in the PPAR pathway, and also *LOC100052888* may be involved in the proliferation of equine myosatellite cells. This revealed a new mechanism for lysine to participate in the cellular metabolic process through ceRNA network at the level of gene regulation, further enriched the understanding of the mechanism by which lysine affects the growth of equine muscle satellite cells and provided a new perspective for an in-depth understanding of the regulation of muscle growth and development and the enhancement of equine athletic performance.

## 5. Conclusions

In this study, it was determined that 0.5 mmol/L lysine promotes the best proliferation of equine myosatellite cells, and analyzed by whole transcriptomics technology, it was found that lysine upregulates *ABRA*, *FHOD3*, *MYOZ3*, *ACTN1*, and *ACTN3* genes to promote the growth and development of skeletal muscle and to improve the performance of horse sprinting. By ceRNA network analysis, *miR-145* was found to act as a target for the negative regulation of the *LOC100052888* gene and to be involved in the PPAR signaling pathway to regulate cholesterol metabolism. Meanwhile, key regulatory genes (*ACADL*, *FADS2*, and *PLIN5*) in the PPAR signaling pathway were upregulated, and identified that lysine mediates the PPAR signaling pathway, regulates lipid metabolism and mitochondrial function, and promotes satellite cell proliferation. The present study reveals the complex metabolic process of satellite cells in equine skeletal muscle after lysine supplementation, which provides an important theoretical basis for the improvement of equine muscle mass and athletic performance.

## Data Availability

In this experiment, the data are included in the article and Supplementary Materials. Further inquiries can be directed to the corresponding author.

## Supplementary Materials

The following supporting information can be downloaded at: https://www.mdpi.com/article/10.3390/ani15121711/s1, Table S1: Raw data for FPKM values of genes.

## Appendix A

**Table A1:** Formulation of culture medium for cell culture.

Culture Solution Name	Culture Fluid Composition
Culture fluid	89% DMEM, 10% FBS, 1% dual antibody
100 mmol/L Lysine culture fluid	0.183 g Lysine powder, 10 mL aseptic and enzyme-free water
Lysine-free DMEN culture solution	84 mg/L Arginine culture fluid, Lysine and arginine free DMEM
Different concentrations of lysine proliferating cultures (0.0, 0.2, 0.5, 1.0, 2.0 mmol/L)	Lysine-free DMEM culture medium, 10%FBS, 1% Dual Antibody, Different volumes (0, 20, 50, 100, and 200 μL) of 100 mmol/L lysine culture medium
84 mg/L arginine culture solution	0.084 g arginine powder, aseptic and enzyme-free water

**Table A2:** Primer information used in this study.

Gene	Primer Sequence (5′→3′)	Product Length (bp)	Annealing Temperatur (°C)
*ACADL*	F:AGGAGTAAGAACAAATGCCAGAAAGG	107	58.58
	R:CCGCCACTACGATCACAACATC		
*PLIN5*	F:CCCACTTCCTGCCCATGACC	80	61.9
	R:TTCCACCGAGCCCACTTCAG		
*SCD5*	F:CGTGGTGCTCATGTGCTTCC	115	59.85
	R:GTTGAGTGAGATGGTATAGCGAAGG		
*FADS2*	F:ATGTGTTCGTCTTGGGTGAATGG	81	58.54
	R:CATGCTGGTGGTTGTAAGGAAGG		
*PAX7*	F:AGAAAGCCAAGCACAGCATCG	98	58.71
	R:CTTCAGAGGGAGGTCGGGTTC		
*KI-67*	F:ACTGTTCTTCATTCTTCAGGACTTCC	120	57.76
	R:CTTCACTTCTCCATTACGGCTCAC		
*MYOD*	F:CCAGCGGGCACCACCAAG	83	63.73
	R:GCGGCGGTCGGCGTTAG		
*GAPDH*	F:GCATCCTGGGCTACACTGAG	111	63.73
	R:AGTGGTCGTTGAGGGCAAT		
*CSPG4*	F:CTTGAGGAGGACGAGTACGAGGAG	144	60.71
	R:GGTGAAGTTGGCGAAGACAGGAG		
*KIF2C*	F:CGGTCTCGCCATCAAGATCCAAC	111	60.79
	R:GCACCTCCTTCTGTCCATTCCAC		
*MATK*	F:CCGAGGCTCTCAAACAAGGGAAG	124	60.6
	R:GACACCTCCTTCAGCGACATCTTG		
*TPX2*	F:CCAGGCAGCAGGAGGAAGAG	123	59.19
	R:ATACGGGCACAGTCAGAGGATG		
*INPP5D*	F:ACTCACCGCTTCACCCACCTC	108	62.69
	R:GTACTGCTGCTGCCTGATCTTCTG		
*PPID*	F:GCAGGCCGCAATACAAATGGTTC	120	59.87
	R:CAGTATCCTTGCCACACCCATTCC		
*UNC5B*	F:CCATCGACCACAACCTCATCATCC	136	60.21
	R:GACCAGCCGCCATTCACATAGAC		
*LOC100069322*	F:GCACTCGTCTAGCCCTCATCATC	119	59.37
	R:CACACTGTAGTCCAGGTCCTCAAG		
